# Enhanced spin orbit interaction of light in highly confining optical fibers for mode division multiplexing

**DOI:** 10.1038/s41467-019-12401-4

**Published:** 2019-10-17

**Authors:** P. Gregg, P. Kristensen, A. Rubano, S. Golowich, L. Marrucci, S. Ramachandran

**Affiliations:** 1 Department of Electrical and Computer Engineering, Boston University, 8 St Mary’s St, Boston, MA USA; 2 OFS-Fitel ApS, Priorparken 680, Brøndby, 2605 Denmark; 3 Dipartimento di Fisica “Ettore Pancini”, Università di Napoli Federico II and CNR-ISASI, via Cintia, 80126 Napoli, Italy; 4 MIT Lincoln Lab, Lexington, MA USA

**Keywords:** Fibre optics and optical communications, Optical materials and structures, Optical physics

## Abstract

Light carries both orbital angular momentum (OAM) and spin angular momentum (SAM), related to wavefront rotation and polarization, respectively. These are usually approximately independent quantities, but they become coupled by light’s spin-orbit interaction (SOI) in certain exotic geometries and at the nanoscale. Here we reveal a manifestation of strong SOI in fibers engineered at the micro-scale and supporting the only known example of propagating light modes with non-integer mean OAM. This enables propagation of a record number (24) of states in a single optical fiber with low cross-talk (purity > 93%), even as tens-of-meters long fibers are bent, twisted or otherwise handled, as fibers are practically deployed. In addition to enabling the investigation of novel SOI effects, these light states represent the first ensemble with which mode count can be potentially arbitrarily scaled to satisfy the exponentially growing demands of high-performance data centers and supercomputers, or telecommunications network nodes.

## Abstract (web summary)

Fiber designs that can that can keep up with increasing data demands are lacking. Here, the authors describe an interaction between the spin and orbital angular momenta of light which enables propagation of 24 states in a single optical fiber with low cross-talk, even in the presence of fiber perturbation.

## Introduction

The spin^1^ (**S**) and orbital^2,3^ (**L**) angular momenta of light can be treated as independent in paraxial optical systems typically encountered in the lab. In such a basis, neglecting the Gouy phase, one can describe propagating optical fields as:

1 $${\hat{\mathbf{E}}}_{l,m}^s\left( {r,\varphi ,z} \right) = {\hat{\boldsymbol{\sigma }}}^se^{il\varphi }F_{l,m}(r;z)e^{i\beta z}$$

Here, ${\hat{\mathbf{\sigma }}}^s = \left( {{\hat{\mathbf{x}}} + is{\hat{\mathbf{y}}}} \right)/\sqrt 2 ,$ for *s* = ±1, are the circular polarizations, $\beta = 2\pi n_{{\mathrm{eff}}}/{\it{\lambda }}$ is the field’s propagation constant, *n*_eff_ is its effective index, *l* and *m* are the azimuthal and radial mode orders, (*r*, *φ*, *z*) are cylindrical coordinates, and *F*_*l,m*_(*r*; *z*) is a function describing the mode’s electric field distribution and whose z dependence includes only beam scaling and phase curvature evolution due to diffraction. In this representation, *l* and s are respectively identified as the photon OAM and SAM (in units of reduced Planck constant, ℏ) along the propagation direction, *z*.

The eigenmodes of optical fibers, which share the cylindrical symmetry of free-space paraxial optical systems, are conventionally described in a similar fashion. Crucial differences, however, are enacted by the transverse confinement of light, analogous to electron confinement in a potential well^4^. Light-matter interaction at dielectric interfaces leads to spin-orbit interactions (SOI)^5^, one of the most well-known manifestations of which is a spin-dependent correction to the modal propagation constants through the photonic Spin Hall Effect^6–8^. For clarity, we use “state” to describe any propagating optical field in free space or fiber, eigenstate to denote a field which is an eigenvector of the z-component of the **S**, **L**, or **J** (total angular momentum) operators, and “mode” to describe a propagation eigenmode in a fiber. Although exact fiber modes can be readily calculated by a brute-force solution to Maxwell’s equations without special consideration of SOI effects, a physically intuitive means to describe this impact of SOI is by expressing the influence of the confinement on the modes’ angular momenta as a first-order correction of the unperturbed (i.e., paraxial free space) modes^9^.

The effect of SOI can be considered in three regimes – negligible SOI, weak SOI, and the rarely- considered strong SOI case. Under the negligible SOI approximation, the solutions are the so-called LP modes, where light is treated as a scalar quantity, analogous to free space beams, and the handedness of OAM and SAM plays no role in determining the modal propagation constants: $\beta = \beta _{\left| l \right|,m}$. Although this approximation is a useful computational tool due to its simplicity, because SOI always exists at some level, mode beating among the LP modes of a given $\left| l \right|$ will be observed in real fibers, leading to the erroneous conclusion that $\left| l \right| \, \ne \, 0$ modes are always unstable in fibers.

Under the weak SOI approximation, first-order degenerate perturbation theory reveals, for $\left| l \right| \, > \, 1$^10^, splitting of the modes into degenerate pairs of spin-orbit aligned (*ls* > 0) and spin-orbit anti-aligned (*ls* < 0) OAM eigenstates, depending on whether the Spin Hall Effect is parallel or antiparallel to the direction of ray rotation. Other known SOI manifestations, such as path-dependent polarization rotations via geometric phases^11,12^ similarly introduce differential phases, but leave the eigenmodes unchanged. Hence, first-order perturbation theoretic analysis of standard optical fibers results in modes which are both **L** and **S** eigenstates, as with paraxial free space systems, but with the degeneracy between spin-orbit aligned and anti-aligned modes broken. Alternatively, using traditional fiber optics parlance, the degeneracy between *HE*_*l+*1_,_*m*_ and ${EH_{{l-1},m}}$ modes is said to be lifted.

SOI-induced effective index splitting, *Δn*_eff_, is small (~10^−6^–10^−5^) in most common fibers. This near-degeneracy is why OAM eigenstates are viewed as unstable in practice, since in-fiber mode coupling is driven by the difference in *n*_eff_ between modes^13^. Only with optical fiber design advancements in the previous decade was it revealed that OAM eigenstates could be long lived in fiber by exacerbating this SOI effect, utilizing annular waveguide designs that yield an order-of-magnitude larger *Δn*_eff_^14,15^. In addition, novel fiber designs such as helically twisted photonic crystal fiber^16,17^, or multicore fibers featuring OAM supermodes^18^, have demonstrated promise. Such OAM-supporting fibers have found myriad applications ranging from nanoscale microscopy^19,20^ to telecom capacity scaling via mode-division multiplexing^21–23^. However, scaling this engineering approach to increasing mode count has limitations, due to the difficulties of enhancing SOI across a wide range of mode orders without introducing a diversity of radial mode orders, which can strongly couple with the desired OAM modes. Practically, the largest ensemble of uncoupled fiber modes achieved to date over any reasonable bandwidth and fiber length (using OAM, or any other basis^24^) is 12^25,26^. A previous paper^27^ has claimed a fiber supporting 36 OAM modes over centimeter-length-scales, but no supporting quantitative data was offered for this claim. In addition, analysis of that reported fiber in view of the theory introduced here, reveals that many of the modes in that fiber could not have been OAM eigenstates.

Here we show that up to 24 electromagnetic modes which are eigenstates of total angular momentum, but which are not eigenstates of either OAM or SAM, can be excited and propagated through a novel thin-ring air core fiber over device lengths (10 m) with high (>93%) purity, resulting in the transmission of the largest number of uncoupled spatial modes in a single-core optical fiber, to date. Such propagating modes have no known analog in free space or weakly guiding fiber optics, and are created by the interplay between a confinement-enhanced SOI and the dielectric anisotropy of the fiber. Our results demonstrate effects of photonic SOI for propagating modes on the micro-scale, counterintuitive to most known SOI manifestations. We expect that this ensemble of unique modes will find applications in expanding the capacity of fiber-optic networks^28,29^ either as a high-capacity transmission medium or at the fiber-device level for realizing high-bandwidth network nodes. Fundamentally, this fiber enables further probing of the SOI of light and related phenomena such as transverse SAM carried by evanescent waves^30^, or acting as a photonic testbed for electronic SOI effects.

## Results

### Strong SOI and perturbation theory

The transition matrix element between a mode ${\hat{\mathbf{E}}}$, with indices (*l*, *s*), and ${\hat{\mathbf{E}}}^{\prime}$ with indices $(l^{\prime} ,s^{\prime} )$, due to the waveguide’s dielectric structure, $\varepsilon (r)$, is given by (see Supplementary Note 2 for derivation):

2 $$\langle{\hat{\mathbf{E}}}^{\prime} (r,\varphi ,0){\mathrm{|}}d\varepsilon {\mathrm{|}}{\hat{\mathbf{E}}}(r,\varphi ,0) \rangle= C\delta _{l^{\prime} + s^{\prime} - l - s}\left\{ {{\int} {rdrF_l\frac{{\partial [{\mathrm{ln}}\;\varepsilon \left( r \right)]}}{{\partial r}}\frac{{\partial F_{l^{\prime} }}}{{\partial r}} - l^{\prime} s^{\prime} } {\int} {rdr\frac{{F_lF_{l^{\prime} }}}{r}\frac{{\partial [{\mathrm{ln}}\varepsilon \left( r \right)]}}{{\partial r}}} } \right\}$$

where *C* is a constant and $\delta _k$ is 1 if *k* *=* *0*, and 0 otherwise, expressing total angular momentum conservation. The second integral term is a direct manifestation of SOI, as indicated by the characteristic $ls$ prefactor. In the negligible SOI region, the left hand side of Eq. 2 is considered to be zero, while in the weak SOI regime, the $l^{\prime} s^{\prime}$ term leads directly to the separation in effective index of the spin-orbit aligned and spin-orbit anti-aligned modes^10^. Enhancing the SOI across a wider manifold of modes in the weak-SOI regime requires exacerbating the effect of this term for many mode orders simultaneously. This requires increasing dielectric constant or confinement, and the attendant increase in modal volume often results in solutions with a multitude of radial mode orders, *m* (see Eq. 1). The problem with the existence of modes with a diversity of *l* as well as *m* values is that this represents modes of differing symmetries, and there exist no systematic physical principles that govern their respective mode separations. In other words, in waveguides that support a multitude of both *l* and *m* modes, accidental degeneracies abound, and even if a careful solution where all modes are sufficiently separated in *n*_eff_ is numerically found, group-velocity dispersion demands that such a solution would be valid for only a narrow range of frequencies^.^

Overcoming this limitation requires accessing the seldom-probed limit of large SOI, where novel behavior arises from a second, subtler, nontrivial element of (2): $l - l^{\prime} = s^{\prime} - s = \pm 2$. Continuing the perturbation analysis, the eigenstates are determined as:

3a $${\hat{\mathbf{E}}}_{ \pm |l|}^ \pm \left( {r,\varphi ,z} \right) = N\left\{ {{\hat{\mathbf{\sigma }}}^ \pm e^{ \pm i|l|\varphi }F_{|l|}\left( r \right) + \frac{\langle{{\hat{\mathbf{E}}}_{ \pm (\left| l \right| + 2)}^ \mp {\mathrm{|}}d\varepsilon {\mathrm{|}}{\hat{\mathbf{E}}}_{ \pm \left| l \right|}^ \pm }\rangle}{{\beta _{|l| + 2}^2 - \beta _{|l|}^2}}{\hat{\mathbf{\sigma }}}^ \mp e^{ \pm i(\left| l \right| + 2)\varphi }F_{\left| l \right| + 2}\left( r \right)} \right\}e^{i\beta _{\left| l \right|, \uparrow \uparrow }z}$$

3b $${\hat{\mathbf{E}}}_{ \pm |l|}^ \mp \left( {r,\varphi ,z} \right) = N\left\{ {{\hat{\mathbf{\sigma }}}^ \mp e^{ \pm i|l|\varphi }F_{|l|}\left( r \right) + \frac{\langle{{\hat{\mathbf{E}}}_{ \pm (\left| l \right| - 2)}^ \pm {\mathrm{|}}d\varepsilon {\mathrm{|}}{\hat{\mathbf{E}}}_{ \pm \left| l \right|}^ \mp }\rangle}{{\beta _{\left| l \right| - 2}^2 - \beta _{|l|}^2}}{\hat{\mathbf{\sigma }}}^ \pm e^{i(\left| l \right| - 2)\varphi }F_{\left| l \right| - 2}\left( r \right)} \right\}e^{i\beta _{\left| l \right|, \uparrow \downarrow }z}$$

**Fig. 1:**
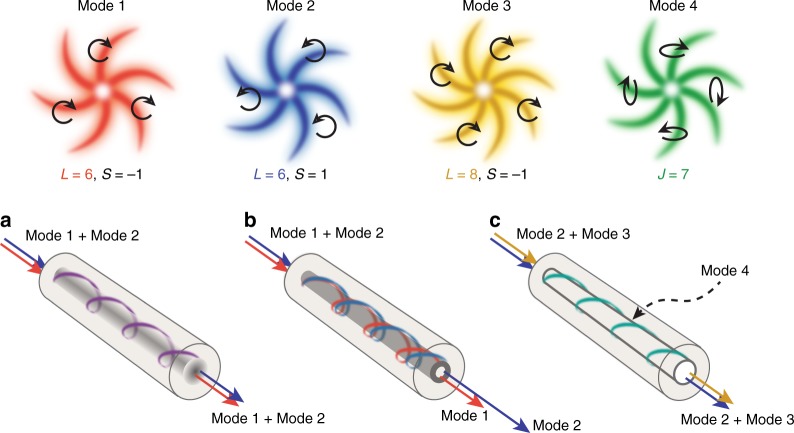
Three regimes of spin orbit interaction. Conceptual illustration of the effect of SOI, with OAM depicted as a spiral (with handedness giving sign, and the number of arms the value of *L*) and SAM as circular black arrows. Colors are used to distinguish different modes and do not indicate the frequency of the light beam. **a** In the scalar approximation (negligible SOI), two OAM states of the same |*L*| will propagate with the same effective index, regardless of polarization. **b** In fiber engineered for OAM-based data transmission, due to SOI, states with OAM and SAM parallel and antiparallel have different effective and group indices. **c** In strongly-SOI-enhanced fibers, superpositions of free space SAM and OAM states with specific relative amplitudes and phases propagate through the fiber as a single mode. These modes are defined by a total AM and can be obtained as the product of a given OAM phase distribution and a space-variant elliptical polarization state (as in mode 4), but they are neither OAM nor SAM eigenstates, and would not be propagation-invariant modes of free space

Here “↑↑” and “↑↓” have been introduced to denote whether the mode is predominantly spin-orbit aligned or spin-orbit anti-aligned, and *N* is a normalization factor. This form of SOI couples modes of the same **J**, but with different **L** and **S** components, resulting in propagating modes which are **J** eigenstates, but neither **L** eigenstates, nor **S** eigenstates^31,32^. Further, this is not solely determined by SOI, but is mediated by the dielectric distribution of the fiber, as shown in Eq. 2, meaning that the magnitude of coupling can be stronger for modes of low *l* than high *l* under certain conditions, which is atypical of SOI effects^5^. Because this coupling is non-resonant, since $\beta _l \, \ne \, \beta _{l \pm 2}$, and is caused by dielectric gradients which are small in most optical fibers, this expression of SOI is typically negligible. The presence or lack thereof of large-SOI modes can also be understood in terms of ray optics, in which the impact of the SOI is explained by the polarization dependence of the Fresnel equations for total internal reflection at an interface^31^, which is negligible in most fibers. Since fractional values of OAM are not propagation invariant eigenstates of free-space^33^, propagating modes of this sort, as suggested by Eq. 3, have no analog in free space optics or weakly guiding fibers. The effect of strong SOI can also be seen as modifying the relative weights of the radially and azimuthally polarized components of a mode, distinguishing the modes of Eq. 3 from traditional *EH* and *HE* vector modes in the weak guidance approximation, or typical free space vector beams^34^ (see Supplementary Note 1). A conceptual illustration of the three regimes: negligible-SOI (scalar), weak-SOI, and strong-SOI, is shown in Fig. 1.

### Characteristics of a thin ring air core fiber

**Fig. 2:**
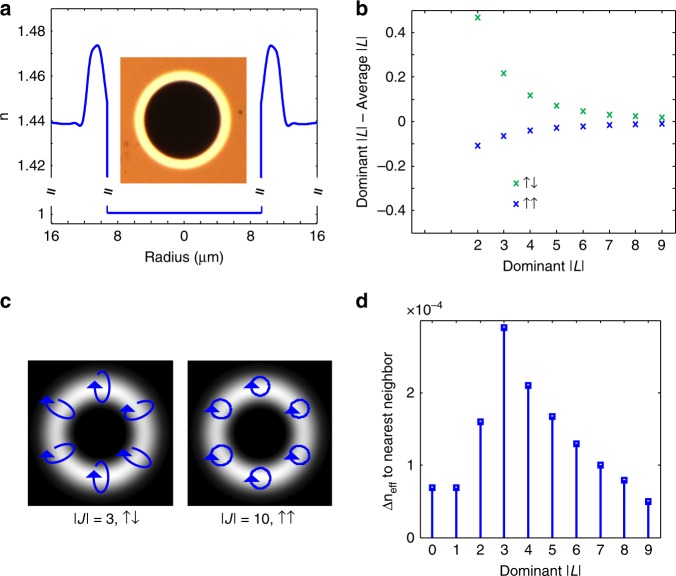
Properties of the thin-ring air core fiber. **a** Measured fiber refractive index profile for thin-ring air core fiber, with cross-section image inset. **b** Calculation, from measured refractive index profile, of effect of SOI in fiber as quantified by the offset in ensemble average OAM from an integer value. **c** Simulated modal electric fields from two representative modes, the |*J*|* = 3*, ↑↓ mode, which strongly feels SOI, and the |*J*| *= 10*, ↑↑ mode, which weakly feels SOI, and is nearly a pure OAM state. **d** Simulated difference in effective refractive index between a mode and its nearest neighbor. From the study of PM fibers, a separatio*n* in *n*_eff_ > 10^−4^ should be sufficient to prevent intermodal coupling due to spin (polarization) effects

A fiber featuring a large air core and a thin high-index ring for modal confinement^35^ is fabricated according to standard modified chemical vapor deposition (MCVD) methods (Fig. 2a). The ring is sufficiently thin that the modes’ electric fields penetrate the air-glass layer, enhancing both the *F* and $\partial \left[ {\ln \varepsilon \left[ {(r)} \right]/\partial r} \right.$ terms in Eq. 2, while also preventing modes of higher radial mode orders. The large radius of the air core forces a large number of modes to encounter the steep air-glass gradient in the refractive index profile, and thus permits macro-scale propagation of modes in a strong SOI regime, which so far has been observed at interfaces^36^, during high numerical-aperture focusing^37^, or in light-matter interaction at the nanoscale^38,39^.

We specify the modes of interest in a simplified form from Eqs. 3a and 3b:

4a $${\hat{\mathbf{E}}}_{ \pm J, \uparrow \uparrow } \propto \left( {A_{\left| J \right|}{\hat{\mathbf{\sigma }}}^ \pm e^{i\left( { \pm J - 1} \right)\varphi } - B_{\left| J \right|}{\hat{\mathbf{\sigma }}}^ \mp e^{i\left( { \pm J + 1} \right)\varphi }} \right)F(r)e^{i\beta _{J, \uparrow \uparrow }z}$$

4b $${\hat{\mathbf{E}}}_{ \pm J, \uparrow \downarrow } \propto \left( {B_{\left| J \right|}{\hat{\mathbf{\sigma }}}^ \pm e^{i( \pm J - 1)\varphi } + A_{\left| J \right|}{\hat{\mathbf{\sigma }}}^ \mp e^{i( \pm J + 1)\varphi }} \right)F(r)e^{i\beta _{J, \uparrow \downarrow }z}$$

Here *J* is the mode’s total angular momentum component along the propagating axis, and, as before, “$\uparrow \uparrow$” and “↑↓” signify whether the mode is predominantly spin-orbit aligned or anti-aligned. In traditional fiber terminology, (4a) would be called *HE*_*J*_,_1_ and (4b) would be called *EH*_*J*_,_1_, but would lack the circular symmetry typically ascribed to these modes in the weak guidance approximation^15^. Due to the strongly confining nature of the waveguide, $F_J\left( r \right) \cong F_{J^{\prime} }\left( r \right)\forall \left( {J,J^{\prime} } \right)$, allowing the definition of a single radial function, *F*(*r*). In the weak-SOI case $\left| {A_j} \right| \gg \left| {B_j} \right|$, with the limit $\left| {B_j} \right| \to 0$, resulting in “standard” OAM modes that are analogous to free-space modes in electric field. In the strong-SOI case, there exist two modes for each |*J*|, and these modes share OAM components. For instance, ${\hat{\mathbf{E}}}_{6; \uparrow \uparrow }$ and ${\hat{\mathbf{E}}}_{6; \uparrow \downarrow }$ both possess OAM components of *L* *=* *5*. We refer to these modes of the same *J* as *SOI pairs*. Note that although these modes share a common total angular momentum, their dominant OAM components (where “dominant component” is defined as the component with largest coefficient, A, which is *L* *=* *J* *−* *1* for ↑↑ and *L* *=* *J* *+* *1* for ↑↓ modes) are separated by 2. These dominant OAM components correspond to azimuthal mode order in the weak-SOI case.

The extent of the SOI for each mode, and hence its departure from well-known OAM eigenstate behavior in paraxial systems, can be quantified by a difference between the dominant OAM and the average OAM per photon, which is by definition $(J - 1)\left| {A_j} \right|^2 + (J + 1)\left| {B_j} \right|^2$ for ↑↑ modes and $(J - 1)\left| {B_j} \right|^2 + (J + 1)\left| {A_j} \right|^2$ for ↑↓ modes, with the normalization $\left| {A_j} \right|^2 + \left| {B_j} \right|^2 = 1$. As evident in Fig. 2b, lower-order modes feature stronger SOI. This is consequent of the lower order modes having higher electric field values at the air-glass interface, due both to having a lower dominant OAM^40^, and a higher effective refractive index, meaning less power in the evanescent tails in the glass cladding. Two exemplary modal electric fields are shown in Fig. 2c. The strongly SOI-impacted |*J*| = 3, ↑↓ mode shows spatially varying elliptical polarization, while the higher order |*J*| = 10, ↑↑ mode has nearly-circular polarization. The difference in effective index between a mode and its nearest (nondegenerate) neighbor, a proxy for mode stability in optical fibers, is seen (Fig. 2d) to decrease as mode order increases. The lowest order modes were not transmitted because they had nearest neighbors overlapping between mode groups. The remaining six mode orders, with 4 modes per order, performed as per the design, and enable a 24-state ensemble for mode-diverse communications. Note that this fiber, although still essentially a ring fiber, supports modes which are dramatically different from the modes of OAM-supporting fibers reported by different groups in the past. The fibers described in ^21,22^ and even ^25^, which also featured an air core, are still well-described in the weak-SOI ($\left| {B_j} \right| \to 0$) regime. However, as discussed previously, scaling mode count in OAM fibers demands enhancing SOI across a wide range of modes. Utilizing a thin-ring approach to avoid multiple radial mode orders requires large index gradients to retain mode volume, which will invariably lead to the angular momentum modes supported in this fiber, rather than OAM modes. For this reason, such modes represent the only known methodology, reported to date, to scale past the currently limited alphabet of mode-mixing-free states available from fibers supporting OAM, or other conventional eigenbases.

### Mode excitation and transmission

**Fig. 3:**
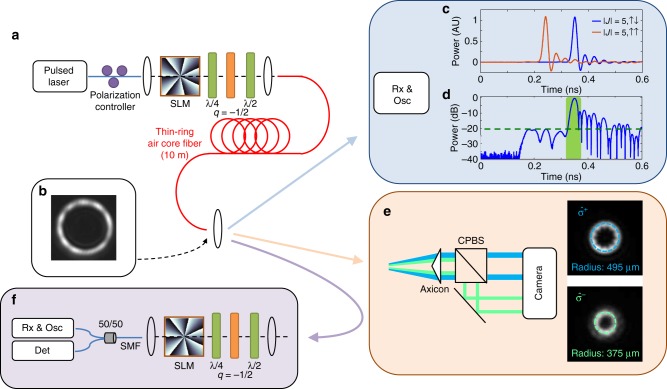
Experimental apparatus for mode excitation and characterization. **a** Experimental apparatus for excitation and characterization of angular momentum fiber modes. **b** Near field image of the fiber under test with a pure *|J|* *=* *5*, ↑↓ mode excited. **c** Two independent time of flight measurements showing selective excitation of SOI pairs *|J|* *=* *5*, ↑↑ and |*J|* *=* *5*, ↑↓, and **d** time of flight measurement of |*J*| *=* *5*, ↑↓ on log scale, showing 20 dB+ suppression of the SOI pair. **e** Measurement of the far field of the |*J*| *=* *5*, ↑↓ mode from (**d**) after splitting in polarization and propagating through an axicon, which converts angles to position. Two different ring sizes, corresponding to different free space OAM states, are observed as expected. **f** Reciprocal setup for transmission matrix measurement

To excite the modes in Eqs. 4a and 4b, which are non-integral combinations of free-space **L** and **S** eigenstates, we utilize the spin-to-orbit conversion properties of *q-*plates^41,42^, as shown in Fig. 3a. The combination of an SLM and *q*-plate, with waveplates before and after the *q*-plate (see Methods for more details), enables controlled tuning across a hybrid-order Poincaré sphere spanned by the two modes in Eqs. 4a and 4b simply by tuning free space waveplates^43^. Since all modes have similar intensity profiles (Fig. 3b), alignment is performed using a 5 ps mode locked laser centered at 1550 nm, with the output of the fiber under test coupled into a standard 50 μm multimode fiber and relayed to a fast receiver and oscilloscope for detection. Input conditions such as fiber position, mirror tilt, and waveplate orientation are adjusted until parasitic peaks due to misalignment are suppressed. When the correct mixture of *L* and *L-2* (of corresponding polarizations, $\hat{\mathrm{\sigma}}^{\mathrm{s}}$) is launched into the fiber, one strong, isolated pulse is observed, and the SOI pair mode is controllably suppressed. Figure 3c shows separate excitation of a *|J|* *=* *5*, ↑↑ and *|J|* *=* *5*, ↑↓ mode, while Fig. 3d shows clear suppression of undesired modes on a logarithmic scale. With a cleanly excited mode, the near field image of the output facet of the fiber reveals a clean ring intensity pattern (Fig. 3b), as expected. By contrast, in the far field, the free-space modes of differing *L* split and the $\hat \sigma ^ \pm$ components of the beam result in two rings of different sizes (Fig. 3e). The temporal trace of Fig. 3c and the far field image of Fig. 3e, respectively, of the output of the fiber underscore the fact that these pure, propagation invariant, non-integer OAM states of light in strong-SOI optical fibers have no analog in bulk media.

**Fig. 4:**
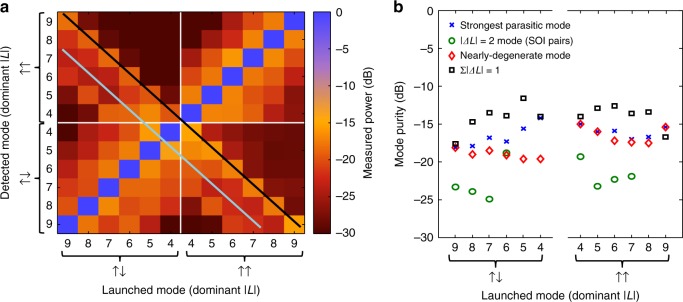
Mode transmission experiment. **a** Transmission matrix over 10 m of thin ring air core fiber including all states of interest. Degenerate states have been collapsed for the sake of simplicity to reduce the matrix from 24 × 24 (shown in Supplementary Fig. 1) to 12 × 12. Parasitic mode suppression is indicated by a strong diagonal trace from lower left to upper right. Undesired modes can be broken into three categories: those which are in-fiber nearest neighbors, which populate the cross-diagonal (black line), SOI-pairs, indicated by a lower cross-diagonal (teal line), and adjacent mode orders ($\left| {\Delta L} \right| = 1$), likely to be excited by system misalignments. **b** Selection of data from mode groups on interest in (**a**). For all modes, the strongest parasitic mode is suppressed by at least 14.2 dB, while the sum of alignment-induced crosstalk (black squares) is typically better than 14 dB. SOI pairs are typically suppressed by more than 20 dB

For quantitative measurements of transmission characteristics, we map out the transfer matrix of a 10- m segment of the fiber under test, bent into loops of radius ~25 cm. One mode is excited, and a mirror-image of the excitation system (Fig. 3f) iteratively projects each mode supported by the fiber onto a single mode fiber (SMF) for detection. By varying the launched mode through all desired modes, a clean diagonal transmission matrix is measured, as seen in Fig. 4a. The transmission matrix is presented here with degenerate states collapsed onto each other for the sake of simplicity (for example, power detected in *J* *=* *5*, ↑↑ and *J* *=* *−5*, ↑↑ is treated as being in the same mode). Such degenerate state coupling, analogous to coupling in conventional polarization division multiplexed systems, could be addressed by digital signal processing or optical pre-compensation. The full 24-mode transfer matrix may be found in Supplementary Fig. 1. A subset of the data of interest is plotted in Fig. 4b. The in-fiber crosstalk, as indicated by the matrix elements along the negative-slope diagonal, is suppressed by 17.7 dB on average, indicating successful suppression of parasitic mode coupling by fiber design. SOI pairs are suppressed to 22.1 dB on average, due to the SOI control exerted in the input coupling system. This is likely not a fundamental limit, and could be improved with more exact waveplates or waveplate control. The worst parasitic mode is suppressed by 16.3 dB on average, and 14.2 dB in the worst case, indicating good overall performance of both the fiber and the coupling system. Matrix elements adjacent to the desired positive-slope diagonal by plus/minus one dominant OAM order, are likely excited by imperfections in the input coupling system, and are summed in Fig. 4b as a proxy for total crosstalk introduced by misalignments and component imperfections in the MUX, which in the worst case, indicates greater than 93% mode purity. We expect these figures to be improved by a more robust input/output coupling system, and that they do not represent a limitation of the fiber under test.

## Discussion

The strong-SOI regime of non-integer OAM modes discussed in this letter has no theoretical limit for scaling the alphabet of available propagating eigenstates of light, given proper choice of materials and fabrication capabilities to enable sufficiently high-index contrast waveguides of the desired size. Hence, we expect the mode count to be substantially more scalable than the record 24 we demonstrated. Mode excitation was achieved with complex, free-space components for our experiments, but solutions to the problem of spatial light shaping between orthogonal mode sets have been demonstrated, hence established designs using multiplane light conversion^44^ or photonic lanterns^45^ can be developed for practical applications using these fibers. This strong-SOI regime is also unique in the sense that it has no known analogies with other systems, such as high-NA focusing, interfaces, and nanowires^46^, where strong-SOI modes manifest, but do not propagate. Moreover, the intriguing topological space provided by these fibers may provide a testbed for probing topics as the angular momenta of evanescent waves, or for studying optical analogs of experimentally inaccessible electronic SOI effects.

## Methods

### Fiber fabrication

The fiber is fabricated using standard modified chemical vapor deposition (MCVD). However, unlike standard fiber draws, the preform is not collapsed on the MCVD lathe. During the draw, the preform is pressurized and nitrogen is passed through the fiber during the draw, which is performed at a few m/s. Equipment used for fabrication is standard production equipment, implying that higher draw speeds should be achievable.

### Fiber characterization

The fiber refractive index profile in Fig. 2a is measured using a commercially available fiber refractive index profiler (IFA-100 Interfiber Analysis), and is measured at 633 nm. Due to the interferometric nature of the measurement, sharp changes in refractive index lead to calculation errors. Thus, the fiber imbibes, via capillary action, refractive index matching oil (Cargille Laboratories) with refractive index close to, but slightly smaller than, the refractive index of Silica to minimize the index discontinuity. In post-processing, the local refractive index is changed to air for values below the index of silica. Simulations were performed with a numerical finite-difference method waveguide solver^47^.

Fiber loss is measured by cutback from 1100 m to ~2 m for each of the guided modes using the pulsed laser source at 1550 nm, with an average mode propagation loss found to be approximately 19 dB/km. Using a white light transmission measurement, we observe strong absorption at 1250 nm and 1380 nm, indicative of water having impregnated the fiber at preform or draw stage. This loss can be improved with better processing. Similar fibers manufactured using revised versions of the same processes have yielded losses as low as 0.8 dB/km^24^.

### Q-plate fabrication

The q-plate device is essentially formed by a thin layer of liquid crystal (LC) having a precise orientation pattern on the transverse plane and sandwiched between two conductive transparent substrates. Two Indium Tin Oxide (ITO) -coated glass substrates form the cell which hosts the LC. Before building the cell, each glass substrate is spin-coated (30 s at 3000 rpm) with a solution of sulphonic azo-dye SD1 (Dainippon Ink and Chemicals) diluted to 1% in dimethylformamide (DMF). The DMF solvent is then evaporated by backing at 120 °C for several minutes. One of the substrates is covered with free-falling dielectric micro-spacers (6 μm average thickness) and the second is placed on top of the first and glued on two sides to obtain an empty two- side-opened LC cell. The orientation pattern is then written on the SD1 azo-dye by photo-alignment, along the direction perpendicular to the optical polarization, by means of a He-Cd laser (~15 mW, 325 nm). The laser polarization is controlled by a half-wave polarizer and sent to a cylindrical lens, which creates a “brush of light” to illuminate only a narrow sector of the cell. Polarizer and sample can rotate on remote-controlled mounts. The typical rotation step is 2°. Exposure times of about 2 h are usually required to obtain the desired pattern. It can be easily shown that the induced topological charge is given by $q = 1 \pm \omega_ {\mathrm{p}}/\omega_ {\mathrm{s}}$, where $\omega_ {\mathrm{p}}$ and $\omega_ {\mathrm{s}}$ are the angular speeds of the polarizer and sample, respectively, and the signs correspond to opposite or same rotation directions of the two mounts. After exposure, the samples are filled with the LC (MLC 6080 mixture from Merck) and sealed by epoxy glue. The cell is finally heated above the LC clearing point to remove occasional LC alignment defects.

### Transmission experiment components and methods

For the time of flight measurements in Fig. 3, we use a passively mode locked picosecond fiber laser (PriTel FFL) with 3 dB bandwidth of approximately 0.6 nm. This is collimated using a reflective collimator, and directed though the series of two Spatial Light Modulators (SLMs) and one q-plate with standard table-top opto-mechanics (Thorlabs), before being coupled into the fiber under test with an f = 8 mm mid-IR AR-coated aspheric lens. Two SLMs are used instead of one to allow for more complex beam shaping in order to create a thin-width high-radius annular beam for fiber coupling. Each SLM adds an OAM (helical phase) and has a lens phase function, as well as an axicon phase function. The SLMs used were Hamamtsu LCOS-SLMx10468-08, with 20 micron pixels. The camera used was an InGaAs Allied Visiion Technologies “Goldeye” camera. The fast detector was a New Focus 1444–50, and the oscilloscope an Agilent Infiniium DCA wide bandwidth scope with detector unit HP83485B (40 GHz electrical).

The collimated beam is directed to the first SLM, and the SLM’s phase pattern is digitally centered on the beam by applying a high topological charge (*L*~10) phase mask and making the output ring beam as uniform as possible. The second SLM is similarly aligned, with the first SLM temporarily set to *L* = 0. The beam after the second SLM is relayed to the fiber coupling lens using two guide mirrors and the beam is aligned into the fiber using time-of-flight measurements to suppress coupling into mode orders which do not have components of the free space OAM state selected by the two SLMs (SOI pairs will not be suppressed at this stage, but component misalignments typically manifest as $\Delta L = 1$ mode excitation). Once this coupling is achieved, the q-plate is added in series, and is centered by observing how the time of flight trace is affected as the q-plate is biased to half-wave retardance and transparency. Unlike in ref. ^32^, the *q* = 1/2 plate is here realized by a *q* = −1/2 plate and a half-wave plate in series. This realization allows for control of both the amplitude and phase of the free space OAM states coupled into the fiber under test (and thus improved mode purity), in which relative amplitude is controlled by the angle of the quarter wave plate, and relative phase by the angle of the half-wave plate. The waveplates, mounted on motorized rotation stages, are set via time domain to minimize coupling into nearest-neighbor (aligned and anti-aligned) modes, and SOI pairs.  Opto-mechanics are sufficiently stable that no adjustment is needed over the course of the measurement. The output projection is aligned similarly, although the input to the fiber under test is iteratively changed to assess and optimize the fidelity of the mode projector.

## Supplementary information

Supplementary Information

## Data Availability

All data related to the experiments described in this manuscript are recorded in laboratory notebooks of members in SR’s research group, and all associated digital data are stored on networked computers at Boston University, whose contents are archived daily. Raw data from Figs. 2–4 available upon request.
