# Supplementary material for: Enhanced spin orbit interaction of light in highly confining optical fibers for mode division multiplexing
Source: Nat Commun. 2019 Oct 17;10:4707. doi: 10.1038/s41467-019-12401-4 (PMC6797754; doi:10.1038/s41467-019-12401-4)
Supplement: Supplementary file 1 — Supplementary Information [file 41467_2019_12401_MOESM1_ESM.pdf]

Enhanced Spin Orbit Interaction of Light in Highly Confining Optical Fibers for Mode Division Multiplexing

Gregg et al.

Supplementary Information

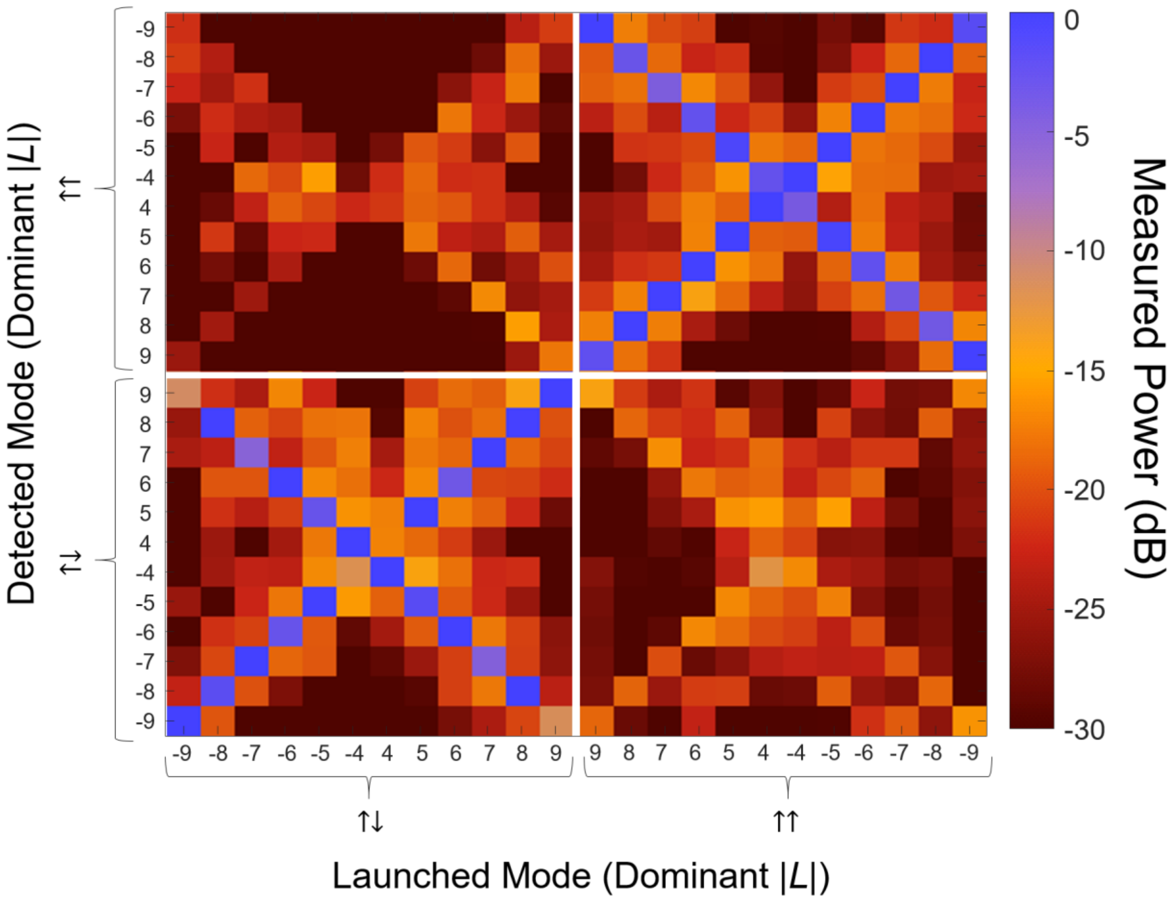

Supplementary Figure 1 | Full mode transmission matrix 24x24 mode transmission matrix for experimental conditions described in manuscript. Each column (launched mode) is normalized with respect to the strongest detected modal component.

Supplementary Note 1: Modal Notation and Conventional Vector Modes

Although the traditional waveguide definition for *HE* and *EH* modes refers to whether the mode is more “TE-like” or more “TM-like” with respect to the longitudinal field components[1], in the fiber community it has also traditionally implied a choice of sines and cosines as azimuthal basis functions instead of complex exponential functions[2]. The spin orbit interaction (SOI) is theoretically nonzero in all fibers, but is trivially small in many standard fibers used for telecommunications. In fibers where the SOI is non-negligible, such as those in[3], it causes a lifting of the degeneracy between $HE_{l+1,m}$ and $EH_{l-1,m}$ modes, but leaves the modal fields unperturbed to a good approximation. Thus, the radial and azimuthal transverse electric field components have nearly identical magnitudes, as shown in **Supplementary Figure 2**. In this regime, conventionally called the “weakly guiding” regime, one can use the basis set of traditional *HE* (*EH*) vector modes, and the basis set of spin-orbit aligned (anti-aligned) OAM modes interchangeably, some apparent advantages of the OAM set, such as ease of free space excitation and a spatially-independent polarization state, notwithstanding.

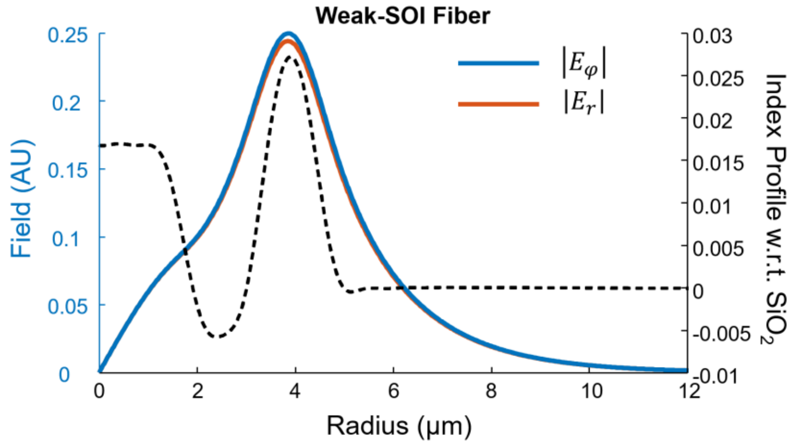

Supplementary Figure 2 | Weak-SOI Electric Field Profiles Transverse electric field profiles for a fiber similar to that shown in [3]. Note that the electric field radial and azimuthal profiles are nearly identical in magnitude, even though this mode is separated from its nearest neighbors in n_{eff} by $\sim 10^{-4}$.

However, in the regime of strong SOI described in this manuscript, the radial and azimuthal electric field components begin to take on different relative magnitudes, causing the pseudo-radial or pseudo-azimuthal polarization states of the mode profiles shown in **Fig. 2** of the manuscript. Two examples, simulated from the fiber under test in the manuscript, are shown in **Supplementary Figure 3**. This change in relative magnitude of radial and azimuthal field components indicates that the modes used in the weak guidance approximation, whether the basis is *HE/EH* modes or spin orbit aligned/anti-aligned OAM modes, are no longer adequate to describe these strong-SOI modes.

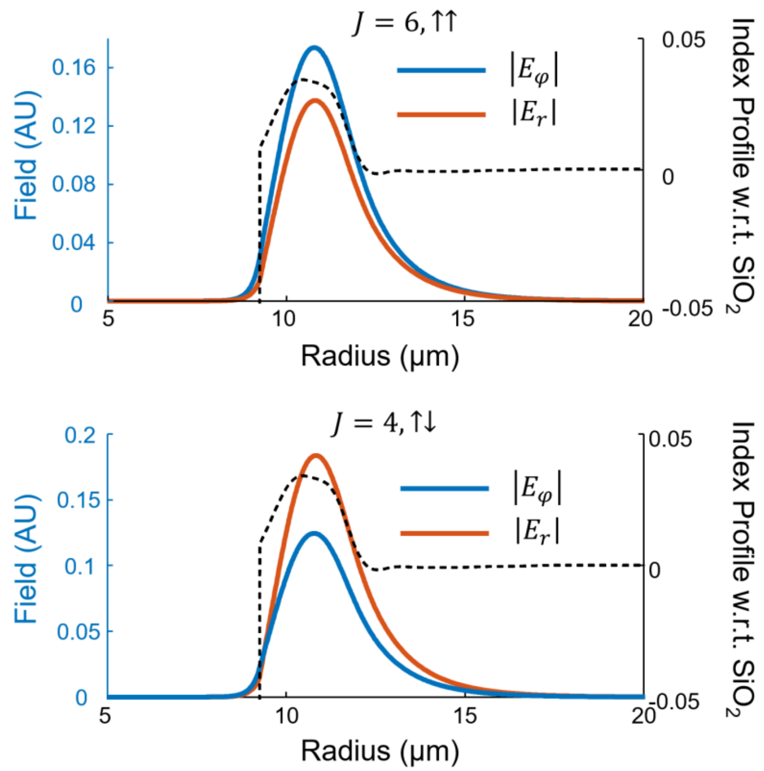

Supplementary Figure 3 | Strong SOI Electric Field Profiles Transverse field distributions for (a) the *J*=6, ↑↑ mode from the thin ring fiber described in the manuscript, and (b) the *J*=4, ↑↓ mode. The obvious difference in radial and azimuthal field components differentiates these modes from those in the "weak guidance" approximation.

Supplementary Note 2: Derivation of Equations (2), (3a), and (3b)

To calculate the effects of the Spin Orbit Interaction according to Perturbation Theory[4], we begin with the scalar vector wave equations (Equations 32-19a and 32-19b from [2]):

$$(\nabla_t^2 + k^2 n^2)\tilde{\Psi} = \tilde{\beta}^2 \tilde{\Psi} \tag{1}$$

$$(\nabla_t^2 + k^2 n^2)\Psi + \nabla_t\{\Psi \cdot \nabla_t[\ln(n^2)]\} = \beta^2 \Psi \tag{2}$$

Here $\tilde{\Psi}$ is the scalar solution with propagation constant, $\tilde{\beta}$, Ψ is the exact vector solution with propagation constant, β, and although no bolding is used, it is understood that all modes and all Laplacians are vector in nature, although $\tilde{\Psi}$ is easily factorable into a vector and scalar part. We introduce a scale parameter, ϵ, assumed small, and expand the vector mode solution and eigenvalue in order of ε.

$$\Psi = \tilde{\Psi} + \epsilon \Psi^{(1)} + \epsilon^2 \Psi^{(2)} + \cdots \tag{3}$$

$$\beta^2 = \tilde{\beta}^2 + \epsilon {\beta^2}^{(1)} + \epsilon^2 {\beta^2}^{(2)} + \cdots \tag{4}$$

We also assume that the perturbation (vector) term in Supplementary Equation (2) is of order ϵ. Substituting Supplementary Equations (3) and (4) into (2), and balancing terms by order in ϵ yields:

No ϵ
$$(\nabla_t^2 + k^2 n^2)\tilde{\Psi} = \tilde{\beta}^2 \tilde{\Psi} \tag{5}$$

ϵ
$$(\nabla_t^2 + k^2 n^2)\Psi^{(1)} + \nabla_t\{\tilde{\Psi} \cdot \nabla_t[\ln(n^2)]\} = {\beta^2}^{(1)} \tilde{\Psi} + \tilde{\beta}^2 \Psi^{(1)} \tag{6}$$

We now make the following assumptions:

(a) Radial mode order m=1 assumed for all modes.

(b) We use the scalar vortex beams as a basis for $\tilde{\Psi}$:

$$\tilde{e}_{|l|,j} = \hat{\sigma}^s e^{il\varphi} F_{|l|}(r) e^{i\beta_{|l|} z} \tag{7}$$

$$\widetilde{\Psi}_{|l|,n} = \sum_j c_{n,j}\,\tilde{\mathrm{e}}_{|l|,j} \tag{8}$$

l is the mode's vortex order and can be positive or negative, *s* denotes the sign of the mode's spin and can be either +1 or -1, and *j* is a dummy index for notational convenience, running from 1-4 for $|L|$>0. In this notation, *s* depends on *j*. Since all OAM modes of a given |*l*| are degenerate in the scalar picture, the scalar solution can be an arbitrary sum of these modes, with weights $c_{n,j}$ in Supplementary Equation (8). The second index, *n*, represents the fact that there must still be 4 orthonormal modes. Supplementary equation (8) is introduced only for notational convenience. Note that for a passive waveguide, $F_{|l|}(r)$ is a real function.

Dot multiplying (S6) with $\tilde{\mathrm{e}}^*_{|l|,k}$, and integrating over 2D space:

$$\int dA\tilde{\mathrm{e}}^*_{|l|,k}\cdot(\nabla_t^2 + k^2n^2)\Psi^{(1)}_{|l|,n} + \int dA\tilde{\mathrm{e}}^*_{|l|,k}\cdot\nabla_t\{\widetilde{\Psi}_{|l|,n}\cdot\nabla_t[\ln(n^2)]\}$$

$$= \beta^{2^{(1)}}\int dA\tilde{\mathrm{e}}^*_{|l|,k}\cdot\widetilde{\Psi}_{|l|,n} + \tilde{\beta}^2_{|l|}\int dA\,\tilde{\mathrm{e}}^*_{|l|,k}\cdot\Psi^{(1)}_{|l|,n} \tag{9}$$

Which simplifies to:

$$\int dA\tilde{\mathrm{e}}^*_{|l|,k}\cdot\nabla_t\{\widetilde{\Psi}_{|l|,n}\cdot\nabla_t[\ln(n^2)]\} - \beta^{2^{(1)}}\int dA\tilde{\mathrm{e}}^*_{|l|,k}\cdot\widetilde{\Psi}_{|l|,n} = 0 \tag{10}$$

Or

$$\sum_j c_j\int dA\tilde{\mathrm{e}}^*_{|l|,k}\cdot\nabla_t\{\tilde{\mathrm{e}}^*_{|l|,j}\cdot\nabla_t[\ln(n^2)]\} - \beta^{2^{(1)}}\delta_{jk} = 0 \tag{11}$$

When repeated for every possible *k*, Supplementary Equation (11) gives a series of equations from which c_j can be solved by taking the determinant of the equation system. This method also

solves for $\beta^{2^{(1)}}$ and lifts the degeneracy among the 4 modes in Supplementary Equation (7). This process is well-known[5], and is not repeated here, but breaks the degenerate between spin-orbit aligned and spin-orbit anti-aligned OAM modes.

If we assume that the first order correction to the wave function of a particular mode from modes outside of its degenerate set of |*l*| is a sum of other fiber modes $l' \neq l$:

$$\Psi_{|l|,n}^{(1)} = \sum_{|l'|,n'} C_{|l'|,n'} \widetilde{\Psi}_{|l'|,n'} \tag{12}$$

Repeating the same procedure as above, and making some mathematical simplifications according to the discussion in Supplementary Reference 2 between equations 32-21 and 32-22, we find:

$$C_{|l'|,n'} = \frac{\int dA(\nabla_t \cdot \widetilde{\Psi}_{|l'|,n'}^{*})\{\widetilde{\Psi}_{|l|,n} \cdot \nabla_t[\ln(n^2)]\}}{\tilde{\beta}_{|l'|}^2 - \tilde{\beta}_{|l|}^2} \equiv \frac{\left\langle E_{|l'|,n'} \middle| d\varepsilon \middle| E_{|l|,n} \right\rangle}{\tilde{\beta}_{|l'|}^2 - \tilde{\beta}_{|l|}^2} \tag{13}$$

Where $d\varepsilon$ is short-hand for the vector perturbation term (unrelated to the scale parameter, ϵ). Combining Supplementary Equations (12) and (13) with (8), and plugging in the known solutions to (11), we can rewrite the vector solution to first order in ϵ as:

$$\Psi_{|l|,n} \approx \hat{\sigma}^s e^{il\varphi} F_{|l|}(r) + \sum_{|l'|,n'} \frac{\left\langle E_{|l'|,n'} \middle| d\varepsilon \middle| E_{|l|,n} \right\rangle}{\tilde{\beta}_{|l'|}^2 - \tilde{\beta}_{|l|}^2} \widetilde{\Psi}_{|l'|,n'} \tag{14}$$

By calculating the transition matrix elements in the right-hand term, we can identify which scalar mode orders contribute to the exact solution. Assuming that the waveguide is rotationally symmetric, using the identities:

$$\hat{r} = \frac{1}{2}\left(\hat{\sigma}^+ e^{-i\varphi} + \hat{\sigma}^- e^{i\varphi}\right) \tag{15a}$$

$$\hat{\varphi} = \frac{1}{2i}\left(\hat{\sigma}^+ e^{-i\varphi} - \hat{\sigma}^- e^{i\varphi}\right) \tag{15b}$$

and evaluating the divergence and gradient operands in cylindrical coordinates, one finds:

$$\begin{aligned}\left\langle E_{|l'|,n'}\middle| d\varepsilon \middle| E_{|l|,n}\right\rangle \\ \propto \int d\varphi e^{i\varphi(l'+s'-l-s)} \\ \times \int r dr \frac{\partial \ln \varepsilon[(r)]}{\partial r}\frac{F_{|l|}}{r}\left(r\frac{\partial F_{|l'|}}{\partial r} - s'l'F_{|l'|}(r)\right)\end{aligned} \tag{16}$$

The azimuthal integral in Supplementary Equation (16) is zero unless the argument inside the exponential is zero, since *s*, *s'*, *l*, and *l'* are integers. Thus, (16) can be reduced to:

$$\begin{aligned}\left\langle E_{|l'|,n'}\middle| d\varepsilon \middle| E_{|l|,n}\right\rangle \\ \propto \delta_{l'+s'-l-s} \int r dr \frac{\partial \ln \varepsilon[(r)]}{\partial r}\frac{F_{|l|}}{r}\left(r\frac{\partial F_{|l'|}}{\partial r} - s'l'F_{|l'|}(r)\right)\end{aligned} \tag{17}$$

Which is exactly Eqn. (2) in the manuscript, with the notational difference of the dummy mode index, *n*, used here instead of the mode's spin, *s*.

Supplementary References

[1] Snitzer, E., "Cylindrical Dielectric Waveguide Modes." *J. Opt. Soc. America* **51**, 491-8 (1961).
[2] Snyder, A.W. and Love, J.D., Optical Waveguide Theory. Chapman and Hall, 1983.
[3] Bozinovic, N., Yue, Y., Ren, Y., Tur, M., Kristensen, P., Huang, H., Willner, A.E., and Ramachandran, S., "Terabit-scale orbital angular momentum mode division multiplexing in fibers." *Science* **340**, 1545-8 (2013).
[4] Das, A., Lectures on Quantum Mechanics. Hindustan Book Agency, New Delhi (2012).
[5] Golowich, S. and Ramachandran, S., "Impact of fiber design on polarization dependence in microbend gratings." *Opt. Express* **13**, 6870-7 (2005).
